# MR sialographic assessment of the masseter muscle and the ductal kinking in patients with recurrent parotitis

**DOI:** 10.1007/s11547-024-01802-1

**Published:** 2024-03-21

**Authors:** Pasquale Capaccio, Matteo Lazzeroni, Francesco Lo Russo, Sara Torretta, Daniele Di Pasquale, Giorgio Conte, Maria Cristina Firetto, Gabriele Nicolino, Michele Gaffuri, Gianpaolo Carrafiello

**Affiliations:** 1 Department of Otolaryngology and Head and Neck Surgery, Fondazione IRCCS Ca’ Granda Ospedale Maggiore Policlinico, Via Francesco Sforza 35, 20122 Milan, Italy; 2 Department of Biomedical, Surgical and Dental Sciences, University of Milan, Milan, Italy; 3 Department of Clinical Sciences and Community Health, University of Milan, Milan, Italy; 4 Neuroradiology Unit, Fondazione IRCCS Ca’ Granda Ospedale Maggiore Policlinico, Milan, Italy; 5 Operative Unit of Radiology, Fondazione IRCCS Ca’ Granda Ospedale Maggiore Policlinico Di Milano, 20122 Milan, Italy; 6 Breast Unit, Fondazione IRCCS San Gerardo Dei Tintori, Via G. B. Pergolesi 33, Monza, Italy

**Keywords:** MR sialography, Masseter muscle, Parotitis, Stensen duct, Bruxism

## Abstract

Dysfunction of the masseter muscle may cause pathological kinking of the parotid duct leading to parotitis; MR sialography is a non-invasive radiological examination that allows to evaluate dynamically the ductal system of the parotid glands. In the present study we aimed to assess the relationships between Stensen’s duct and masseter muscle and their implications in the aetiopathogenesis of recurrent parotitis secondary to masseter muscle dysfunction. Forty-one patients with recurrent unilateral parotitis and nine with bilateral recurrent parotitis, all with a clinical suspicious of masseter muscle hypertrophy due to bruxism were enrolled. They underwent ultrasonography as a first line examination and then MR sialography and sialendoscopy. Different anatomical features were studied. Involved parotid glands had a wider duct compared to contralateral unaffected parotid glands of patients with recurrent parotitis (*p* = 0.00134); male subjects with parotitis had a longer duct compared to the salivary glands of healthy patients (*p* = 0.00943 for affected glands and *p* = 0.00629 for the contralateral). A concordance between the evidence of an acute duct angle during sialendoscopy and a wider duct in patients with parotitis was observed although not statistically significant. These initial findings suggest that the masticatory muscle dysfunction related to bruxism seems to condition alteration of parotid duct course and anatomy thus favouring the occurrence of recurrent parotitis. A specific diagnostic iter based on clinical evaluation, dynamic ultrasonography and MR sialography, is therefore, mandatory to confirm the relationship between masseter muscle anatomy and parotid duct anomalies; this is the premise for an adequate therapeutic approach to underlying masticatory muscle disorder.

## Introduction

Recurrent parotitis is mainly caused by sialolithiasis, strictures and salivary duct anomalies, immune and autoimmune disorders, *ab estrinseco* compression by lymphadenitis or masseter muscle hypertrophy [1, 2]. Stensen’s duct leaves the gland from its antero-superior border, crosses over the masseter muscle and then pierces at almost a right angle the buccal fat pad, the buccopharyngeal fascia and the buccinator muscle to open into the oral cavity [2–4]; the fibres of the buccinator muscle wrap Stensen’s duct wall to participate in its peristaltic contraction, thus favouring the flushing out of saliva [3]. Enlargement of the masseter muscle may cause pathological kinking of the parotid duct leading to sialadenitis [4]. Different techniques have been proposed for the evaluation of recurrent parotitis, both radiological, such as sialography and MR sialography, and non-radiological such as sialendoscopy.

Conventional sialography is still considered a valid imaging modality to explore salivary duct system [5] although related to some limitations such as the use of contrast media or radiation, the dependence on the operator’s technical skills for successful ductal cannulation, and the risk of displacement of distally placed ductal stones. It is also contraindicated during acute salivary inflammation and overfilling with contrast material may result in a false-positive diagnosis of sialectasis [6, 7]. MR sialography, instead, is a hydrographic technique in which static or slowly flowing fluids in the body such as saliva are imaged as high signal intensity structures against a dark background with very low signal intensity. It is therefore a non-invasive exam useful for the evaluation of parenchyma and the ductal system of salivary glands [7, 8].

Sialendoscopy, introduced in clinical practice since the 90 years [9], is a minimally invasive technique for the direct exploration of the duct system [10]. Diagnosis of recurrent parotitis secondary to masseteric muscle dysfunction should be considered every time diagnostic sialendoscopy does not reveal, in a patient with a history of bruxism, intraluminal causes of obstruction, such as sialolithiasis, duct stenosis, polyps or more rarely foreign bodies, but only ductal kinking and mucous plugs [10]. The evidence of an acute ductal angle (the so-called duct wall beyond the angle) should be, therefore, interpreted as a possible sign of the *ab estrinseco* compression of the enlarged masseter muscle [4].

The anatomy of the masseter muscle has not been precisely defined and various classification methods have been proposed for the muscle’s hypertrophy in which the most frequently used parameter is muscle thickness measurement [11]; Xie et al. [12] have classified masseter muscle hypertrophy into 5 types (minimal, mono, double, triple, excessive) depending on its bulging clinical features. In particular, masseter muscle hypertrophy is a condition caused by an overuse of the jaws due to clenching, bruxism or other temporomandibular joint disorder, it has a higher occurrence between the ages of 10 and 40 years, it can have either a unilateral or bilateral presentation and can present with facial swelling or pain [13]. Masseter muscle dysfunction may be unilateral or bilateral in the affected individuals. In case of bilateral masseter muscle dysfunction patients can have square angled lower face appearance; when it is unilateral, it may cause facial asymmetry [14]. Patients with recurrent parotitis secondary to masseter muscle dysfunction typically manifest swelling and parotid pain during breakfast, after a night of clenching and bruxism.

Regarding Stensen’s duct anatomy only little information is available on its length and diameter in patients with or without a history of parotitis [15, 16]. No study has yet analysed the anatomical relationship between masseter muscle and Stensen’s duct in patients with recurrent parotitis; we investigated, by means of MR sialography, the metrical and morphological relations between those two structures and their implications in the aetiopathogenesis of recurrent parotitis secondary to masseter muscle dysfunction.

## Material and methods

### Subjects

Forty-one patients with a history of recurrent unilateral parotitis (median age 47.5 years; IQR: 37–53.5 years, female *n* = 22) and nine with bilateral parotitis (median age 54 years; IQR: 38–67 years, female *n* = 9) and suspect of masseter dysfunction due to bruxism were enrolled at the ENT department of Fondazione IRCCS Ca’ Granda Ospedale Maggiore Policlinico of Milan between January 2017 and February 2022. This study was performed in line with the principles of the Declaration of Helsinki and was approved by the local ethics committee.

All the patients initially underwent power Doppler ultrasonography to exclude stones and duct anomalies and blood examinations to exclude immune and autoimmune disorders; dynamic MR sialography was then performed followed by diagnostic sialendoscopy. Patients with megaducts, defined as a dilatation of Stensen’s duct > 10 mm [17] as well as patients with salivary stones, autoimmune parotitis or neoplastic conditions were excluded from the study.

**Fig. 1:**
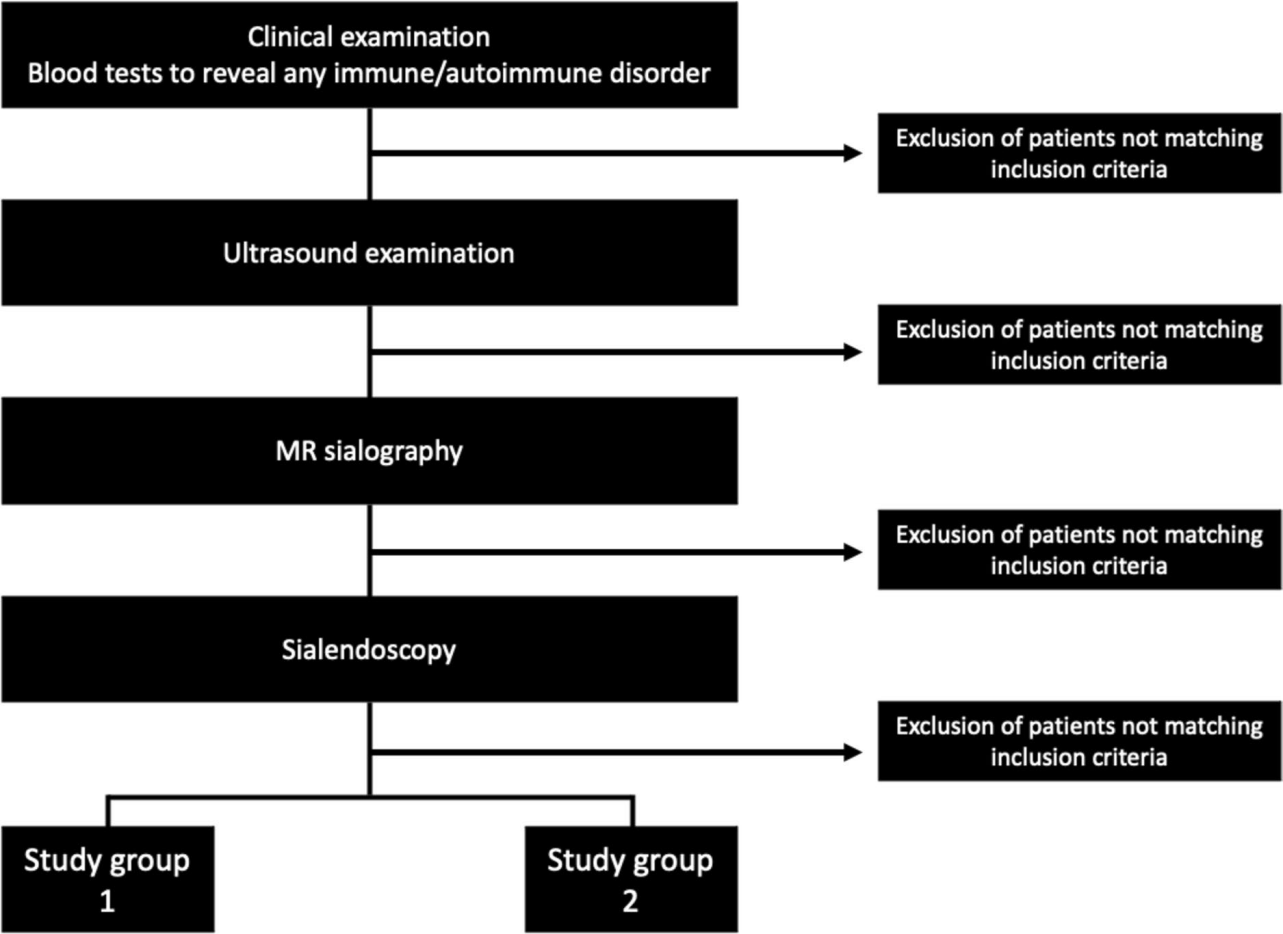
Flow chart showing inclusion and exclusion criteria for patients with recurrent parotitis

The parotid glands of the selected patients were then split into two groups, thus dividing the affected glands from the symptom-free salivary glands of patients with a history of recurrent parotitis (Fig. 1):

- Study group 1: patients with recurrent parotitis; measurements taken on the affected parotid gland. In case of bilateral sialadenitis each parotid gland was considered separately.
- Study group 2: patients with recurrent parotitis; measurements taken on the contralateral unaffected parotid gland.

For the control group, instead, patients who had undergone MR sialography for recurrent submandibular sialadenitis were selected; control group patients did not suffer from any immune/autoimmune disorder and did not have a clinical history of recurrent parotitis or abnormal radiological findings, such as megaducts, concerning the parotid glands. A total of 33 control subjects were, therefore, enrolled for the present study (median age: 48 years IQR: 40–59 years; female *n* = 16).

### Clinical examination

Clinical examination was performed in all patients with a history of bruxism including inspection and palpation of the masseter on resting and clenching. Episodes of parotitis mainly occurred early in the morning and near breakfast time.

### Ultrasonography

Ultrasonography (US) was performed by means of a small-parts US transducer (Hitachi H 21, 7.5 MHz) without a standoff device, and colour Doppler US was used to differentiate vascular structures from the salivary ducts. Basal and dynamic (with lemon juice stimulation) images of salivary glands were acquired in order to exclude intraluminal causes of obstruction and to reveal possible mild dilations of the duct system [18, 19].

### MR sialography and anatomical measurements

Dynamic MR sialography (Siemens AG 2015 Software NUMARIS/4 Version syngo MR E11) was performed: sequences were acquired before and after the administration of 3–5 cc of lemon juice to the patients, who were given the recommendation not to swallow until the end of the procedure.

**Fig. 2:**
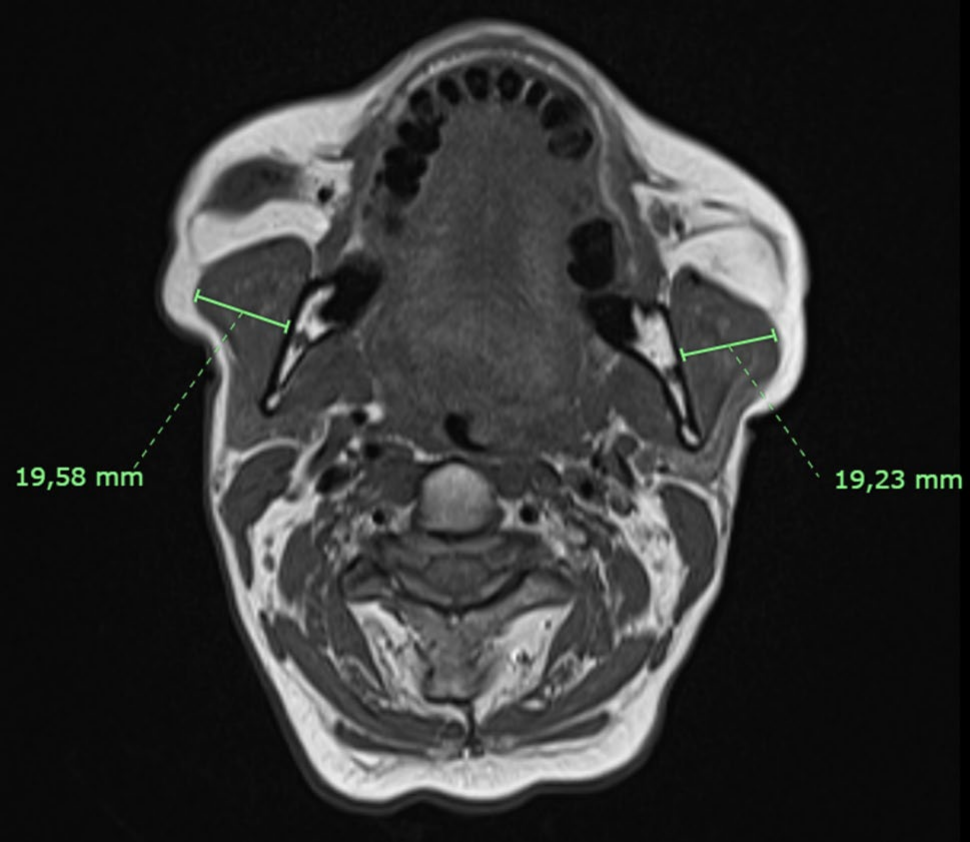
The maximum diameter of the masseter muscle in a patient with bilateral recurrent parotitis

**Fig. 3:**
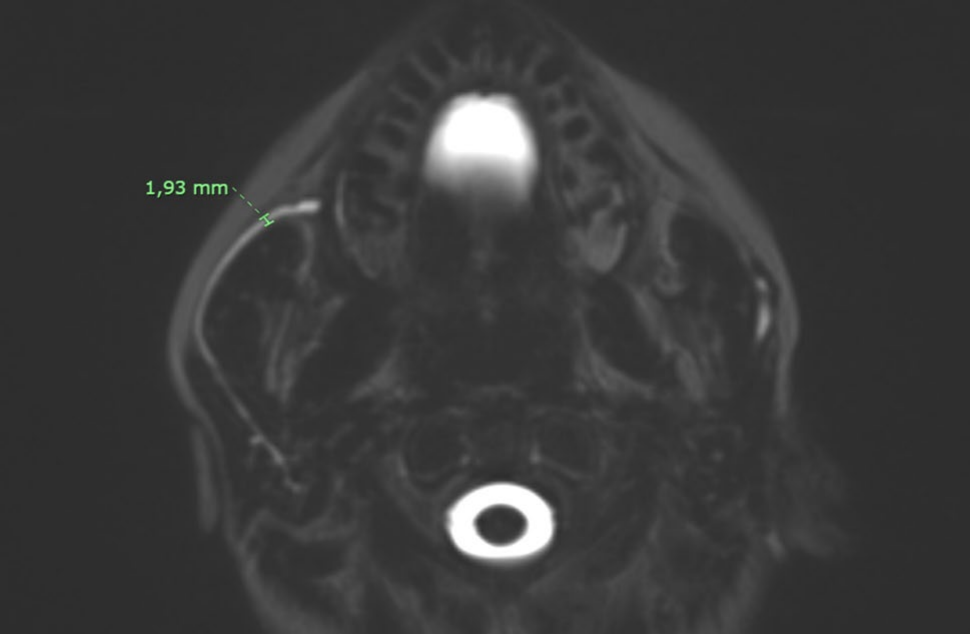
Maximum diameter of the distal third of the Stensen’s duct in a patient with right recurrent parotitis

**Fig. 4:**
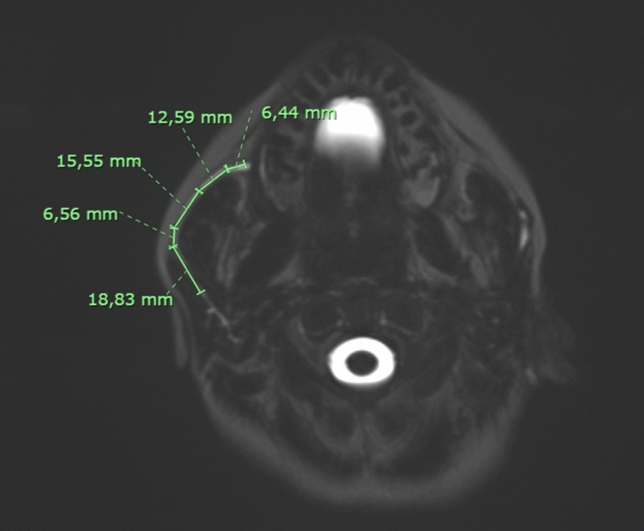
Measurements of the length of Stensen’s duct in a patient with right recurrent parotitis

**Fig. 5:**
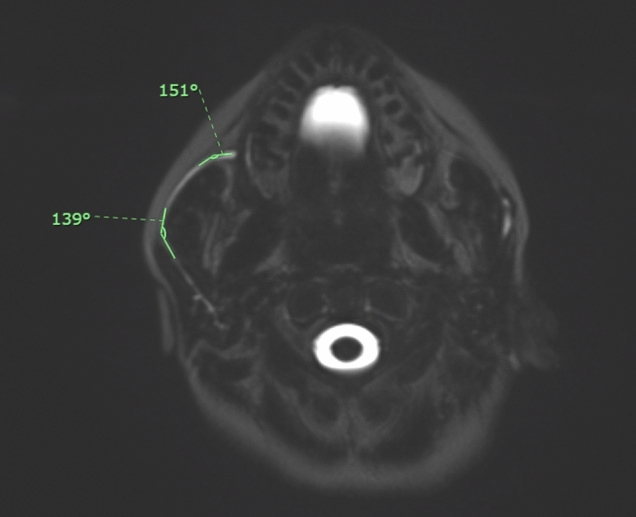
Measurement of the angles of Stensen’s duct when it pierces the buccinator muscle and when it crosses over the masseter muscle in a patient with right recurrent parotitis

For each subject the following parameters were acquired:

- Masseter thickness, the maximum diameter of the masseter muscle on axial MRI images (Fig. 2);
- Parotid duct diameter, the maximum diameter of Stensen’s duct measured on MR sialography images after stimulation with lemon juice (Fig. 3);
- Parotid duct length, the overall length of Stensen’s duct from its origin from the merging point of the secondary branches to its end in the papilla after stimulation with lemon juice (Fig. 4);
- 1° angle before and after stimulation, the angle of Stensen’s duct when it pierces the buccinator muscle before and after stimulation with lemon juice (Fig. 5);
- 2° angle before and after stimulation, the angle described by Stensen’s duct when it crosses over the masseter muscle before and after stimulation with lemon juice (Fig. 5).

### Sialendoscopy

Sialendoscopies were performed in the operating theatre by an expert salivary surgeon and by a resident in training. All sialendoscopies were performed under local anaesthesia and sedation. Firstly, Stensen’s duct ostium was dilated with a blunt-tip lacrimal probe dilator (Karl Storz Co., Tuttlingen, Germany), then a 0.8 mm semiflexible angled sialendoscope (Erlangen sialendoscope, Karl Storz Co., Tuttlingen, Germany) was inserted in the duct ostium and passed through the duct system up to the tertiary ductal branches thanks to saline solution continuous irrigation; Sialendoscopies were considered concluded when all the viable branches were explored, pervious and free from mucous plugs [20].

### Statistical analysis

The statistical analysis was performed using R 4.1.2 [R Core Team (2021) R: A language and environment for statistical computing. R Foundation for Statistical Computing, Vienna, Austria. URL https://www.R-project.org/]. A *p* value smaller than 0.05 was considered significant.

Shapiro–Wilk tests were performed to test the distribution normality of the continuous variables. Since the assumption of normality was violated, the Kruskal–Wallis test was used to compare the continuous variables amongst groups, and a Chi-square test was used to compare nominal variables. p values of univariate analysis were corrected using the Bonferroni method. Dunn–Holm’s tests were carried out for post hoc pairwise comparisons.

We performed an intraclass correlation analysis (ICC) using a two-way mixed effect model and “average of 2 rater” unit to assess reliability of parotid imaging metrics. Mean estimations along with 95% confidence intervals (CI) were reported for each ICC. Interpretation was as follows: < 0.50, poor; between 0.50 and 0.75, fair, between 0.75 and 0.90 good; above 0.90, excellent.

## Results

Study group 1 included 59 parotid glands, Study group 2 included 41 parotid glands and the Control group 66 parotid glands. Groups differed in terms of gender (*χ*2(2) = 7.93, *p* = 0.019), being subjects with bilateral sialadenitis all females. The Kruskal–Wallis did not show a statistically significant difference in terms of age amongst subjects’ groups, (*z*(2) = 0.939, *p* = 0.625).

**Fig. 6:**
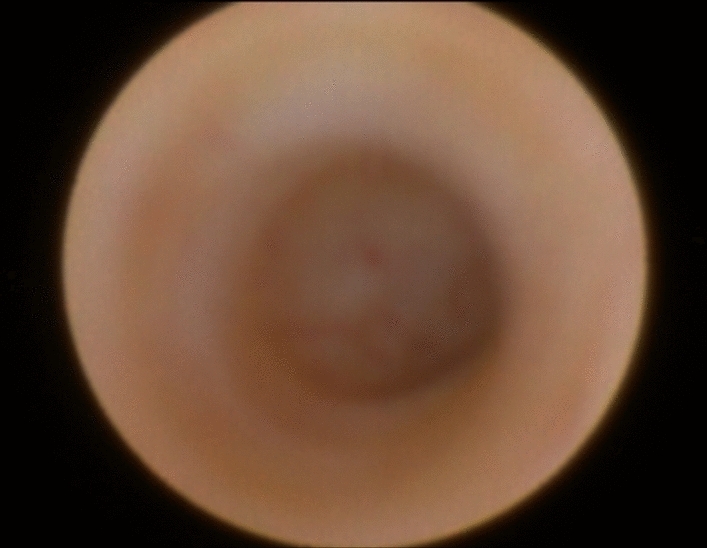
Sialendoscopic finding of an acute ductal angle of the Stensen’s duct (the so-called ductal wall) corresponding to the mark of the masseter muscle

Ultrasonography did not reveal dilatation of Stensen’s duct or the presence of any detectable cause of obstruction such calculi; hypoechoic feature of the parenchymal texture as well as increased vascularization of the parenchyma was visible in most of the involved glands. Diagnostic sialendoscopy revealed an acute duct angle in the middle third of the main duct (“the ductal wall”, in Fig. 6) that made it difficult to explore the rest of the salivary system; only some mucous plugs were detected.

We analysed separately each subjects’ parotid sialo MR imaging data, as summarized in Table 1. Considering the whole subjects’ parotids cohort, there was no statistically significant difference in terms of parotid imaging metrics. A subgroup analysis by gender, showed that contralateral unaffected parotids (study group 2) of male subjects with sialadenitis and affected parotids (study group 1) of male subjects with sialadenitis had a longer duct length compared to parotids of male control subjects (respectively, *p* = 0.00943 and *p* = 0.00629; Table 2). There was no statistically significant difference for female subjects’ parotids metrics (Table 3).

**Table 1:** Univariate analysis of the variables involved in the development of sialadenitis. Numeric variables are expressed as median and interquartile range (in parenthesis)

	Control group	Study group 2	Study group 1	*Z*	Effect size	*p*
Total number	67	40	59			
Masseter muscle thickness (mm)	17 (15–18.5)	16.5 (15–19.2)	15 (15–19)	0.47	0.00941	1
Parotid duct diameter (mm)	1.5 (1.1–1.8)	1.2 (0.9–1.8)	1.55 (1–6)	4.89	0.018	0.552
Parotid duct length (mm)	59 (54.2–61)	62 (56–65)	60 (56.5–63)	6.50	0.028	0.234
First Angle before stimulation (°)	146 (135–153)	144 (137–155)	148 (137–155)	0.67	0.0084	1
First Angle after stimulation (°)	144 (134–152)	148 (140–152)	145 (139–154)	2.24	0.0015	1
Difference in the first angle after and before stimulation (°)	− 1 (− 6–2)	0 (− 6.5–5)	0 (− 5–4)	1.54	0.003	1
Second Angle before stimulation (°)	144 (136–152)	145 (139–149)	143 (130–149)	0.911	0.008	1
Second Angle after stimulation (°)	144 (138–154)	146 (140–152)	145 (138–151)	0.331	0.0125	1
Difference in the second angle after and before stimulation (°)	3 (0–8)	2 (− 0.25–5.75)	1 (− 4–4)	0.849	0.009	1

*n* = frequencies; mm = millimetres

**Table 2:** Univariate analysis of the variables involved in the development of sialadenitis for male subjects. Continuous variables are expressed as median and interquartile range (in parenthesis)

	Control group	Study group 2	Study group 1	*Z*	Effect size	*p*
Total number	34	17	17			
Masseter muscle thickness (mm)	17 (15–18)	19 (17–21)	19 (16–22)	8.34	0.0976	0.0924
Parotid duct diameter (mm)	1.5 (1.08–1.82)	1.4 (1–1.9)	5.7 (1.4–7)	7.70	0.0876	0.128
**Parotid duct length (mm)**	**59 (54.2–61)**	**63 (59.5–68.8)**	**62 (60–70.5)**	**13.5**	**0.176**	**0.0072**
First Angle before stimulation (°)	143 (136–151)	148 (142–154)	144 (132–156)	1.1	− 0.0138	1
First Angle after stimulation (°)	144 (136–154)	145 (140–152)	144 (133–158)	0.0186	− 0.0305	1
Difference in the first angle after and before stimulation (°)	0 (− 4–3)	− 2 (− 9.75–0.25)	1 (− 1–4)	3.39	0.0214	1
Second Angle before stimulation (°)	145 (138–152)	141 (136–147)	143 (127–146)	2.48	0.0101	1
Second Angle after stimulation (°)	149 (141–156)	145 (136–146)	145 (136–152)	2.75	0.0160	1
Difference in the second angle after and before stimulation (°)	3 (0–7.5)	− 1 (− 4–3)	8 (6–8.5)	2.56	0.0119	1

	Control group vs. study group 2	Control group vs. study group 1	Study group 1 vs. study group 2
Parotid duct length	0.00943 **	0.00629 **	0.880

Bold, asterisk values indicate statistically significant result

*n* = frequencies; mm = millimetres

Post-hoc analysis with Dunn’s test of the imaging metrics involved in the development of sialadenitis for male subjects. *p* values are corrected with the Holm’s method

**Table 3:** Univariate analysis of the variables involved in the development of sialadenitis for female subjects. Numeric variables are expressed as median and interquartile range (in parenthesis)

	Control group	Study group 2	Study group 1	*Z*	Effect size	*p*
Total number	33	23	42			
Masseter muscle thickness (mm)	17 (15–19)	15 (14–17)	15 (14–17.8)	4.69	0.0283	0.56
Parotid duct diameter (mm)	1.5 (1.2–1.7)	1.15 (0.9–1.65)	1.2 (1–5)	2.57	0.00603	1
Parotid duct length (mm)	59 (54.8–61)	61 (54–63.5)	59 (56–62)	0.315	− 0.0177	1
First Angle before stimulation (°)	148 (135–153)	144 (136–154)	149 (139–154)	0.891	− 0.0117	1
First Angle after stimulation (°)	143 (132–149)	148 (139–152)	146 (140–153)	3.54	0.0163	1
Difference in the first angle after and before stimulation (°)	− 3 (− 7.5–1.25)	2 (− 3–5.25)	− 1 (− 5–4)	4.40	0.0253	0.666
Second Angle before stimulation (°)	140 (132–150)	146 (141–151)	143 (131–150)	1.66	0.0052	1
Second Angle after stimulation (°)	143 (135–148)	147 (143–154)	145 (138–151)	2.45	0.0076	1
Difference in the second angle after and before stimulation (°)	3 (0–8.5)	2 (2–7)	− 2.5 (− 5.5–0.5)	3.81	0.0301	1

*n* = frequencies; mm = millimetres

Considering subjects with unilateral sialadenitis, a pairwise comparison showed that parotids with sialadenitis had a wider duct compared to contralateral unaffected parotids (*p* = 0.00134; Table 4).

**Table 4:** Univariate paired analysis of the parotid metrics involved in the development of sialadenitis. Continuous variables are expressed as median and interquartile range (in parenthesis)

	Study group 1*n* = 41	Study group 2*n* = 41	*Z*	Effect size	*p*
Masseter muscle thickness (mm)	16.5 (15–20)	16.5 (15–19.2)	188	0.281	0.128
**Parotid duct diameter (mm)**	**2 (1–5)**	**1.2 (0.9–1.8)**	**564**	**0.514**	**0.00134**
Parotid duct length (mm)	60.5 (58.2–64.8)	62 (56–65)	220	0.00531	0.965
First Angle before stimulation (°)	147 (137–152)	144 (137–155)	366	0.200	0.248
First Angle after stimulation (°)	144 (138–150)	148 (140–152)	193	0.0761	0.604
Difference in the first angle after and before stimulation (°)	− 1 (− 4.75–2.75)	0 (− 6.5–5)	214	0.0761	0.703
Second Angle before stimulation (°)	142 (128–148)	145 (138–149)	84	0.295	0.172
Second Angle after stimulation (°)	144 (138–151)	146 (139–153)	124	0.0139	0.961
Difference second angle after and before stimulation (°)	4 ( − 3–9.75)	− 0.5 (− 3–6.5)	144	0.204	0.339

Bold values indicate statistically significant result

*n* = frequencies; mm = millimetres

A complete concordance between the evidence of an acute duct angle during sialendoscopy and a wider duct in patients with parotitis was observed although not statistically significant. ICC for inter-rater reliability was between fair and excellent for the imaging metrics.

## Discussion

Recurrent parotid swelling is a condition that can arise not only from intrinsic conditions such as stones, ductal stenosis and anomalies, juvenile recurrent parotitis, allergic and autoimmune disorders [1, 2], but also from extrinsic disorders such as lymphadenitis, neoplasia and masseter muscle hypertrophy [4]; in particular areas of tenderness, ligaments, tendons and muscular bands of masseter muscle of patients with bruxism can be the main cause of salivary obstruction due to external compression of the salivary duct system [13, 21].

Fifty patients with unilateral (41 patients) and bilateral (9 patients) parotitis with suspect of masseter muscle dysfunction due to bruxism underwent clinical, radiological (ultrasonography and MR sialography) and sialendoscopic assessment.

First of all, masseter muscle thickness was measured in all patients and control group and was correlated with the results of a systematic review by Reis Durao et al. [22] where muscle thickness, ranging from 12 and 17.2 mm, was reported to be greater in men and elderly individuals. Higher values of muscle thickness were observed in our study groups and control group although not statistical significant (see Table 2), thus suggesting a negligible role of muscular thickness in favouring occurrence of recurrent parotitis. Interestingly, Radsheer et al. [23] observed thickness of the masseter muscle decreases significantly with age in both sexes.

Stensen’s ducts of the parotid glands of male affected subjects were significantly longer than the ones of control subjects (*p* = 0.009 and *p* = 0.006 for study group 2 and study group 1, respectively). Little information is available in the current literature on the anatomy of Stensen’s duct: in a study conducted by Zhu et al. [15] Stensen’s duct mean length in healthy subjects was shown to be 52.0 mm, while in affected subjects 58.0 mm. Horsburgh et al. [16] in a series of sialographies measured a mean length of Stensen’s duct of 52.0 mm for healthy patients and 53.0 mm in affected patients. Our measurements are slightly higher than the ones found in the literature; a possible explanation has to be found in the type of imaging measurements: as unique studies [15, 16] were conducted on sialographies, the major drawback is that it may create a distorted projection and image that differs from true anatomical size.

Moreover, Stensen’s duct diameter was significantly greater in study group 1 than study group 2 (*p* = 0.001) as confirmed by the MR images done after stimulation with citric acid. Our measurements were overall in accordance with the ones given by previous reports: a study by Zenk et al. [24] on 25 cadavers of patients without a history of salivary pathology showed that mean values of Stensen’s duct diameter were 1.4 mm, while Horsburgh et al. [25] showed mean values of 1.6 mm.

A difference between duct angles in the parotid gland affected by recurrent parotitis and contralateral normal one and control group was observed during dynamic MR sialography although not statistically significant. According to the results of the study, factors other than those anatomical variations observed in the study such as elongation of the Stensen’s duct and increase in duct diameter seem to condition acute angles observed during sialendoscopy in patients with recurrent parotitis. In this regard, we recently demonstrated an impaired tone of the masseter muscle ipsilateral to the affected parotid gland at electromyographic and kinesiographic neuromuscular investigation [4]; the orthodontic treatment with a neuromuscular removable orthosis resulted in the complete resolution of the recurrent parotid symptoms and significant decrease in electromyographic values. As a consequence a recurrent dynamic dysfunction of the masseter muscle together with an anatomical modification of the Stensen’s duct observed at MR sialography may justify the occurrence, early in the morning, of episodes of parotitis in patients who clench teeth during the night. In this regard, gnathologic assessment should be part of the diagnostic work up in patients with recurrent parotitis due to bruxism. A possible association between the angles formed by the confluence of the accessory parotid duct and the Stensen’s duct has been also advocated [15, 16] but without a clear relationship with recurrent parotitis.

## Conclusion

To the best of our knowledge, this is the first paper studying the relationship between the course of Stensen’s duct and masseter muscles by means of a comparative ultrasonographic, MR sialographic and sialendoscopic assessment in patients with recurrent parotitis secondary to masseter muscle dysfunction due to bruxism. Dynamic MR sialography is helpful in detecting minimal modification of the course and diameter of the Stensen’s duct that cannot be revealed by traditional ultrasonography; at the same time MRI capture after stimulation with acid citric may simulate the increase in salivary stimulation occurring in parotid glands early in the morning in concomitance with breakfast time. Elongation of the Stensen’s duct and increase in duct diameter observed at MR sialography in patients with recurrent parotitis do not justify alone the occurrence of parotitis. A multidisciplinary approach based on radiological, otolaryngological and gnathological assessment, is therefore, mandatory to reveal all anatomical and dysfunctional features associated with recurrent parotitis secondary to bruxism.
